# Kernohan Woltman notch phenomenon caused by subdural chronic hematoma: Systematic review and an illustrative case

**DOI:** 10.1016/j.amsu.2022.104006

**Published:** 2022-06-14

**Authors:** Abdelkouddous Laaidi, Saad Hmada, Abdessamad Naja, Abdelhakim Lakhdar

**Affiliations:** NEUROSURGERY Department, University Hospital Center IBN ROCHD, Casablanca, Morocco

**Keywords:** Kernohan woltman notch phenomenon- subdural chronic hematoma- ipsilateral deficit -anticoagulant

## Abstract

Kernohan Woltman Notch Phenomenon (KWNP) is caused by a supratentorial lesion pressing the contralateral cerebral peduncle against the free edge of the tentorium of the cerebellum. It is manifested by neurological signs of ipsilateral localization; cerebral MRI is the most sensitive examination for KWNP. Our patient is a 50-year-old woman, operated in 2011 for aortic and mitral valve replacement by mechanical prosthesis, under oral anticoagulant, consults for headaches evolving for 20 days without any notion of head trauma with installation of a progressively worsening left hemibody deficit. Glasgow coma scale was 14 (E3 V5 M6) with left anisocoria 4mm left/2mm right with left hemiplegia. CT shows a chronic left hemispheric subdural hematoma 13.5mm thick with subfalcorial and ipsilateral temporal involvement of the deficit. The cardiovascular examination as well as the biological assessment was unremarkable. The patient was operated on with a total recovery in 12 days, the anticoagulant is resumed on day 20 postoperatively, with close monitoring. KWNP may contribute to misdiagnosis in patients with bilateral corticospinal tract lesions, and anticoagulation poses a problem in stopping and restarting treatment due to the risk of bleeding on one side and thrombosis on the other side.

## Highlights

• This article presents a review of literature of Kernohan Woltman Notch Phenomenon due to a subdural chronic hematoma.
• The clinical examination revealed a hemiplegic motor deficit associated with consciousness disorders and the CT scan confirmed the diagnosis.
• The relationship between the hypersignal of the cerebral peduncle on MRI and the severity of the clinical picture as well as the delay in postoperative recovery.

## 1 Introduction

**Fig. 1:**
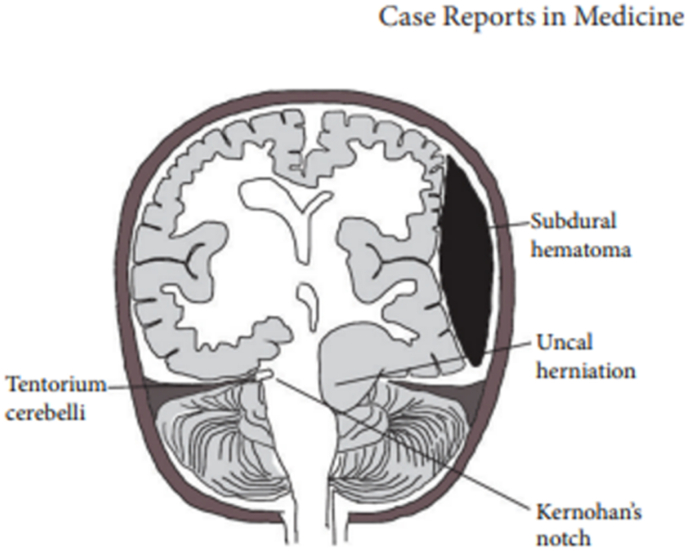
Schematic representation of KWNP. Demonstrating here a subdural hematoma and uncal herniation on the same side. Notching of the midbrain is seen on the opposite side. This damages the contralateral pyramidal tract fibers in the midbrain and causes hemiparesis on the side of subdural hematoma. (6).

Kernohan-Woltman notch phenomenon (KWNP) is defined as compression of the cerebral peduncle against the tentorial edge caused by the displacement of the brain tissue at the ipsilateral side of the paradoxically supratentorial localized lesion which produces ipsilateral hemiplegia or hemiparesis [1]. Is usually seen in patients with brain tumors and severe head injuries [2] (see Fig. 1).

Since their original description in 1929 [3], few patients with Kernohan's notch phenomenon associated with chronic subdural hematoma have been reported in the literature [4].

We report the case of Kernohan's notch phenomenon in a patient with chronic subdural hematoma on anticoagulant treatment.

We conducted a systematic literature review to aggregate all previously reported KWNP. Finally, we discuss the management of this rare phenomenon at the light of the previously reported cases.

## 2 Materials and methods

### 2.1 Systematic review

The PubMed/Medline, Google Scholar, Cochrane library, clinicaltrials.gov, and clinicaltrialregister.eu databases were searched using the following search algorithm: *“*Kernohan-Woltman notch phenomenon” and “chronic subdural hematoma” taking into consideration all articles up to august 2021. All titles and abstracts were verified by two neurosurgeons (AL and SH) to exclude all non-pertinent studies. Articles reporting pediatric patients, patients with acute subdural hematoma, epidural hematoma and cerebral tumors were all excluded.

Studies involving animals or without available full text were also excluded. The references of the selected studies were subsequently searched to identify any additional related articles.

## 3 Results

### 3.1 Case description

We report the case of a 50-year-old woman with a past medical history of aortic and mitral replacement by mechanical prosthesis operated in 2011 with anticoagulant medication. And no history of fall. Presenting with tree weeks of headaches, and two days of the hemi-body heaviness.

Neurological examination on admission demonstrated a Glasgow Coma Scale (GCS) score of 14 (E3, V5, M6). Left pupil was of 4mm, and was nonreactive, right pupil was of 3mm. With left hemiplegia grade 2 on the Medical Research Council (MRC) grading system.

The preoperative CT scan revealed a 13.5mm thick left hemispherical chronic subdural hematoma with falcor and ipsilateral temporal involvement of the deficit.

The patient was operated on the same day. An emergency left trephination was performed to evacuate the chronic subdural hematoma. After the operation, the neurological signs, including deterioration of mental state, left-sided hemiplegia and unresponsive dilated left pupil immediately returned to normal. Third day evolution, motor function had improved to 4/5 on the MRC grading scale and she was discharged with GCS 15 and no cognitive deficits. The anticoagulant is resumed on day 20 postoperatively, with close monitoring. Complete recovery was noted one month after surgery.

### 3.2 Literature review

Twelve cases of Kernohan-Woltman notch phenomenon due to subdural chronic hematoma have been reported including our case and have been reviewed and summarized in Table 1.

**Table 1:** Age varied between 42 and 88 years old with a mean age of 65 years old. A male predominance was noted 67% (8 males, 4 females). Brain CT scan was performed for 10 patients while brain MRI in 2 cases 0.1 Burr hole in 7 cases, craniotomy in 3 cases, 2 burr holes in 1 case. Favorable development was observed in all patients but with variable duration.

Author	Age	Initiale GCS	Pupils	Presention	Surgery	Findings of imaging	Outcomes description	outcomes
Itoyama 1995 [5]	69	NA	NA	left-sided hemiparesis	BH	Right cerebral peduncle: Deformity, no signal change	Left hemiparesis improved significantly postoperatively	Improving
Yasuyuki 2002 [6]	62/M	8	Right dilated pupil		craniotomy	left cerebral peduncle pressed against the free edge of the tentorium (	Complete recovery in the next 25 days	
Bhatoe [7] 2005	56/M	11	Right dilated pupil		BH	Right cerebral peduncle: T2: hyperintense	Gradual recovery in 3 months	Improving
Moon30 2006 [4]	70/M	9			BH	Right cerebral peduncle: T1: hypointense T2: hyperintense	Some remaining hemiparesis postoperatively	Improving
Moon30 2006 [4]	56/F	GCS 11	Fixed and dilated right pupil		BH	No abnormalities at cerebral peduncle	All neurological symptoms immediately resolved	postoperatively Resolved
Fareed 2007 [3]	42/F	GCS 9	Right dilated pupil		mini craniotomy	-Mass effect over the brain parenchyma -Midline shift of 12 mm towards the left side	Complete recovery in the next 2 days	partial weakness of third cranial nerve
Derakhshan 2009 [8]	76/M	GCS 15	Normal		No surgery	No mass effect	–	–
Albayrak 2012 [1]	88/M	GCS 14	Right dilated pupil		BH	Narrowing at left cerebral peduncle, no signal changes	Complete recovery in early post-operative period	Resolved
Sasikala 2014 [9]	60/M	altered sensorium.	Left dilated pupil		BH	hyperdense collection with layering over left fronto-temporo-parietal region with mass effect and midline shift	Complete recovery in the next 2 days	Resolved
Çabalar 2014 [10]	43/M	13	Right dilated pupil		BH	Left cerebral peduncle: T2: hyperintense	Gradual recovery 3 months post-operatively, mRS 4	Improving
Yasuyuki 2002 [11]	62/M	8	Right dilated pupil		Craniotomy	hyperdense collection with layering over left fronto-temporo-parietal region with mass effect and midline shift	Complete recovery in the next 2 days	resolved
Panikkath 2013 [12]	69/F	Comatose			Craniotomy	Shift of the midbrain to the left with hyperintensity in the midbrain in the region of compression	Recovered consciousness	
Our patient 2020	50/F	14	Left dilated pupil		BH		Recovery in 12 days	resolved

This case has been reported in line with the 2020 SCARE guidelines [13].

## 4 Discussion

### 4.1 Key results

KWNP due to a chronic SDH are rare, with only 12 cases, including the present one, reported. They usually present after the sixth decades, with a slight male predominance. They present as a chronic subdural hematoma on MRI or CT scan. Complete surgical removal led to clinical recovery in most cases.

### 4.2 Interpretation

Kernohan's phenomenon is a rare and interesting complication secondary to intracranial mass effect. Since this first description, there have been many reports describing clinical and radiological features of this phenomenon. (1) Kernohan and Woltman have described in 1929 that any supratentorial-localized mass may compress the contralateral cerebral peduncles at the tentorial edge. (2) Petechial hemorrhages may be seen through gradient echo MRI, and cytotoxic edema could also be present. (1).

In the classic ipsilateral brainstem compression by medial temporal lobe herniation from an ipsilateral compressive lesion, the Kernohan Woltman notch phenomenon occurs when brain displacement results in compression of the contralateral crus cerebri by the tentorial edge. (4) We are describing the occurrence of this phenomenon in association with a chronic subdural hematoma.

Hemiparesis can be found in up to 58% of cases of CSDH and mostly the deficit is contralateral to the lesion (direct pressure on the cerebral hemisphere). (10) In rare circumstances the focal neurological deficit can be ipsilateral to CSDH (7) which define the KWNP.

As though, there is no comprehensive clinical trial concerned with the incidence of KWNP, nevertheless, there is limited number of cases of KWNP secondary to CSH [[4], [6], [8]]. We have done a literature revue of all cases of KWNP including our case and found 12 cases.

When the mechanism of Kernohan-Woltman notch phenomenon was considered, we conclude that remarkable brain atrophy was a facilitating factor in development of KWNP secondary to wide subdural hematoma and it was noticeable also in our case. The clinical evaluation is the gold standard in diagnosis of Kernohan-Woltman notch phenomenon. (2).

However, some authors believe that the pathophysiology of KWNP involves the mechanism of cytotoxic edema for which diffusion weighted imaging (DWI) could be more helpful in the initial assessment of KWNP. (4) It shows a hypersignal of the contralateral cerebral peduncle, which is an essential factor to analyze on the imaging and which allows to predict the clinical evolution of the patients. We noticed that the evolution of the patients with a hypersignal of the cerebral peduncles had a slower improvement than those who did not have, which is confirmed by the study of Itoyama and al (9). The signal change shown on MRI due to Kernohan's notch phenomenon causing a permanent tissue damage in the crus cerebri, predicts persistent motor deficit. However, with the gradual onset of a chronic subdural hematoma, Kernohan's notch phenomenon may develop with only transient compression of the crus cerebri. In this situation recovery is more complete than in those patients with signal changes in the crus cerebri identified by MRI. (5).

## 5 Conclusions

Kernohan's phenomenon is a rare false localizing sign, which can lead to diagnostic and clinical confusion. We have reviewed pathophysiology, neuroimaging features associated with this phenomenon due to subdural chronic hematoma and demonstrated that, whilst functional outcome is determined by the lesion of cerebral peduncle, motor function can be regained in most cases. Patients affected by KWNP should be counselled on the rehabilitation potential of the affected limbs, however, more data into the reversibility of KWNP can help to further understand rehabilitation potential.

## Financial disclosure

The authors declared that this study has received no financial support.

## Provenance and peer review

Provenance and peer review Not commissioned, externally peer-reviewed.

## Ethical approval

Written informed consent for publication of their clinical details and/or clinical images was obtained from the patient.

Ethical approval has been exempted by our institution.

## Sources of funding for your research

None.

## Author contribution

Abdelkouddous LAAIDI: writing the paper. Saad HMADA: Corresponding author. Abdelhakim LAKHDAR: Correcting the paper. Abdessamad NAJA: Correcting the paper.

## State the trial registry number – ISRCTN

None.

## Research registration unique identifying number (UIN)

ResearchregistryXXXX

## Guarantor

Saad HMADA, Abdelkouddous LAAIDI.

## Declaration of competing interest

The authors declare having no conflicts of interest for this article.
